# Recombinant protein Ag85B-Rv2660c-MPT70 promotes quality of BCG-induced immune response against *Mycobacterium tuberculosis* H37Ra

**DOI:** 10.3389/fimmu.2025.1430808

**Published:** 2025-03-13

**Authors:** Jiangshan Ouyang, Shaohua Guo, Zhiming Hu, Ting Cao, Jun Mou, Xinxia Gu, Chunxu Huang, Jie Liu

**Affiliations:** ^1^Laboratory of Infectious Diseases and Vaccine, West China Hospital, West China School of Medicine, Sichuan University, Chengdu, China; ^2^Department of Healthcare Intelligence, University of North America, Fairfax, VA, United States

**Keywords:** *mycobacterium tuberculosis*, BCG, vaccine, Ag85B, Rv2660c, MPT70, Th1 cytokines, multi-functional T cells

## Abstract

**Introduction:**

Bacillus Calmette-Guérin (BCG), the only licensed vaccine against *Mycobacterium tuberculosis (Mtb)* infection, has been extensively used worldwide for over 100 years, but the epidemic of tuberculosis (TB) remains a major challenge to human health and well-being. The quest for a more effective vaccination strategy against the *Mtb* infection continues. Boosting the protective immunity induced by BCG with recombinant protein is a feasible approach to improve the efficacy of BCG, due to the proven safety and effectiveness of recombinant proteins as vaccination regimes against a variety of infectious diseases. While being shown to be promising in clinical trials in preventing *Mtb* infection, data suggest this strategy requires further improvement.

**Methods:**

In this study, we developed a novel fusion of proteins derived from major antigenic components of *Mtb*, including Ag85B, Rv2660c, and MPT70 (ARM), and assessed its antigenicity and ability to boost BCG efficacy in a murine model.

**Results:**

The results demonstrated that the ARM immunization induced antigen-specific T and B cell responses and reduced the *Mtb* H37Ra burdens in the lungs and spleen. Mice that were primed with BCG and boosted with the ARM mounted a Th1-type immune response, characterized by an increased proportion of multi-functional ARM- and *Mtb* lysate-specific CD4+ T cells that produced IFN-γ, TNF-α, and IL-2 compared to BCG alone, and reduced the *Mtb* burden without the development of severe lung pathological inflammation.

**Discussion:**

The results of our study demonstrate that the ARM boost improves the quality of the BCG-induced immune response, increases its potency of pathogen reduction, and offers an additional option for enhancing the efficacy of BCG vaccination.

## 1 Introduction

Tuberculosis (TB), caused by the bacterium *Mycobacterium tuberculosis* (*Mtb*), is one of the deadliest human diseases and presents a significant health, social, and economic burden worldwide. Approximately one quarter of the world’s population is infected with the *Mtb*. While the majority of those infected with the *Mtb* maintain a state of latent tuberculosis infection (LTBI) and show no signs of disease (1), approximately 10% of them develop TB disease (2). The World Health Organization reported 10.8 million cases of active TB and 1.25 million deaths from TB in 2023 (3). Bacillus Calmette-Guérin (BCG), a live attenuated strain of *Mycobacterium bovis*, is the only licensed vaccine to prevent the disease and provides significant protection in children, with 58% efficacy against pulmonary TB and 80% efficacy against disseminated TB, such as meningeal and miliary TB (4). However, the efficacy against pulmonary TB varies from 0-80% in adolescents and adults, who are the main source of the TB transmission (5). The current vaccination strategy is insufficient to facilitate the elimination of TB and achieve the goal of ending the epidemic by 2030. One hundred years after BCG becoming available, a quest for a new TB vaccine continues.

Attempts to develop a novel vaccine to replace BCG or an alternative strategy to boost the efficacy of BCG have been explored extensively. Studies have suggested that protein-based boosters may have the potential to enhance the protective effect of BCG (6). Mice primed with BCG and boosted with H56, a recombinant fusion protein of Ag85B-ESAT 6-Rv2660c, showed a robust T cell response, with an increased proportion of multifunctional CD4^+^ T cells and a significant reduction in *Mtb* load compared with mice immunized with BCG alone (7). Guinea pigs primed with BCG and boosted with ID93, a recombinant fusion protein of Rv1813-Rv2608-Rv3619-Rv3620, or GamTBvac, a recombinant fusion protein of Ag85A, ESAT6, and CFP10, showed prolonged survival compared with those immunized with BCG alone although the *Mtb* loads were not significantly or only slightly reduced (8, 9). All these H56, ID93, and GamTBvac immunization strategies are in clinical trials. The data from preclinical studies and clinical trials have suggested that the boosting strategy with recombinant proteins has the potential to improve the efficacy of BCG vaccination. However, the H56, ID93, and GamTBvac have not yet been demonstrated successful in augmenting and sustaining the BCG-induced protection in humans.

To explore an approach enhancing the immune response induced by BCG, we have designed and constructed a recombinant fusion protein, ARM, which selectively incorporates immunogenic components shared by *Mtb* and BCG, including Ag85B, Rv2660c, and MPT70. The Ag85B is a mycolyltransferase engaged in mycobacterial cell wall synthesis and is actively expressed during the bacilli proliferation (10). As an immunodominant antigen, Ag85B induced a robust Th1-type immune response in mice, with increased proportions of memory/effector phenotype and multi-cytokine-producing CD4^+^ and CD8^+^ T cells, CD80 and CD86 costimulatory molecule-expressing antigen-presenting cells, and increased Th1 cytokine production, which enhanced the *Mtb* clearance at similar or higher levels compared with BCG (11). Rv2660c is highly expressed in order to adapt to nutrient starvation during latent *Mtb* infection (12). CD4^+^ T cells isolated from LTBI patients produced high levels of IFN-γ in response to Rv2660c stimulation *in vitro* compared with those from active TB patients, suggesting the role of Rv2660c-induced immunity in preventing the progression of LTBI to active TB (13). Furthermore, Rv2660c facilitated the differentiation of CD4^+^ T cell into multi-functional cytokine-producing cells and the elimination of *Mtb* in mice when combined with Ag85B-ESAT-6. This demonstrated the ability to control LTBI reactivation and to reduce the burden of *Mtb* in mice (7). MPT70 is a secretory protein that is actively expressed when the *Mtb* is in a state of starvation (12) or in the phagosomal environment (14). The MPT70 induced IFN-γ production of peripheral blood mononuclear cells from both active TB and latent TB patients (15), and enhance the protective immunity induced by fusion protein Rv1886-Rv3478-Rv3619 against *Mtb* (16). We anticipated that the combination of Ag85B, Rv2660c, and MPT70, which are preferentially expressed at stages of *Mtb* infection and with proven immunogenicity, would enhance the BCG-induced immune response.

It has been demonstrated that T cell responses participate in the containment of *Mtb* infection. The Th1-type CD4^+^ T cell response played a pivotal role in preventing *Mtb* infection by secreting cytokines such as IFN-γ, TNF-α, and IL-2. The IL-2 has been shown to promote the survival of *Mtb*-specific T cells and the development of immune memory (17); IFN-γ has been demonstrated to induce the production of antimicrobial molecules by macrophages, including oxygen free radicals and nitric oxide (18); TNF-α has been observed to stimulate the migration of effector cells to the site of infection and to induce the production of reactive nitrogen intermediates, which act in synergy with IFN-γ to reduce bacterial burden of *Mtb* (18). In addition, CD4^+^ T cells expressing two or more cytokines were more effective in controlling *Mtb* infection than those expressing a single cytokine (19). In addition to the pivotal role of the cellular immunity, antigen-specific antibodies may also contribute to the host defense against *Mtb* infection by mediating macrophage phagocytosis, complement activation, and enhancing NK cell cytotoxicity (20). For example, the mycobacterial capsular polysaccharide arabinomannan (AM)-specific IgG enhanced macrophage phagocytosis and inhibited mycobacterial growth through FcR-mediated phagocytosis, and this inhibition correlated with the quantity of IgG production (21). The AM-specific IgG altered the transcriptional profile and lipid metabolism, thereby modulating the fitness and susceptibility of *Mtb* to host defense (22). In a clinical trial, the presence of Ag85A-specific IgG was associated with a reduced risk of TB in BCG-vaccinated infants (23). A vaccination regime that elicits a Th1-type T cell response with multifunctional CD4^+^ T cells and a *Mtb*-specific antibody response has the potential to enhance the efficacy of BCG vaccination.

Protein glycosylation and conformation are critical for antigenicity. In BCG-immunized guinea pigs, deglycosylated Ala-Pro-rich antigen had decreased potency in eliciting T cell immune responses (24). Ag85B is glycosylated proteins in their native forms, and the glycosylation are essential for their structural integrity and antigenicity (25, 26). In order to maintain the glycosylated conformation and the antigenicity, we used *Pichia pastoris* (*P. pastoris*) yeast to express recombinant *Mtb* proteins. The *P. pastoris* expression system has been used for expression of heterologous proteins in large quantities and with appropriate glycosylation (27). A study showed that the Ala-Pro-rich antigen produced by *P. pastoris* displayed comparable mannosylation patterns, including glycosylation sites and glycan length, to those by *Mtb* (28). Meanwhile, we used unmethylated cytosine phosphate-guanine oligodeoxynucleotide (CpG ODN) to enhance the potency of Th1-type immune response induced by the ARM. CpG ODN has been found to promote the Th1-type cellular response through Toll-like receptor 9 (29). Here, we evaluated the ARM in terms of antigenicity, induction of T and B cell responses, and its impact on BCG efficacy to assess its potential as a booster to improve the efficacy of BCG vaccination, and to guide the future investigation.

## 2 Materials and methods

### 2.1 Mycobacterium strains and mice

*M. bovis* BCG Pasteur 1173P2 strain and *Mtb* H37Ra strain were purchased from Shanghai Gene-Optimal science and technology (Shanghai, China), and maintained on Middle brook 7H11 agar medium (BD Biosciences, USA). Specific pathogen-free (SPF) female C57BL/6 mice, aged 4-6 weeks, were purchased from Experimental Animal Center of Sichuan University (Chengdu, China) and Chengdu Dossy Experimental Animals (Chengdu, China), and housed under pathogen-free conditions in the Animal Biosafety Level-2 (ABSL2) facility at the Experimental Animal Center, West China Hospital, Sichuan University, China, without cross-ventilation. The animal experiment protocol was reviewed and approved by the Institutional Animal Care and Use Committee (IACUC), West China Hospital of Sichuan University (No. 20220228076).

### 2.2 ARM recombination design

The nucleic acid sequence of *ag85b* (Gene ID: MT682677.1), *rv2660c* (Gene ID: 887222), and *mpt70* (Gene ID: 887724) were downloaded from the National Center for Biotechnology Information (NCBI, USA) database. The sequences encoding the secretion signal peptides of Ag85B, Rv2660c, and MPT70 were identified respectively using the Gram-positive program in SignalP - 5.0 (https://services.healthtech.dtu.dk/service.php?SignalP-5.0); the sequences encoding the transmembrane helices of the Ag85B, Rv2660c, and MPT70 were identified respectively using TMHMM - 2.0 (https://services.healthtech.dtu.dk/services/TMHMM-2.0/). After removal of the identified secretion signal and transmembrane helices fragments, the sequences of *ag85b, rv2660c, and mpt70* were linked in tandem with flexible linkers (GGTGGTGGTGGTTCT)_3_. The tandem sequence was finally optimized for expression in *P. pastoris* using program ExpOptimizer (https://www.novoprolabs.com/tools/codon-optimization).

### 2.3 Recombinant *P. pastoris* construction and expression of ARM

The fragment of the recombinant *arm*, with a length of 1,653 bp, was synthesized and cloned into the vector pUC57 to generate pUC57-ARM. PCR primers for amplification of the *arm* were designed to incorporate partial sequences of pGAPZαA, sites for the restriction enzyme *EcoR* I (forward primer) or *Not* I (reverse primer), and *arm*. 6 × *his* tags were inserted between the sites for restriction enzyme and the partial *arm*. A termination codon was added to the reverse primer. The sequences of the forward and reverse primers are ARM-F, 5’-AGAGAGGCTGAAGCTGAATTCCATCATCATCATCATCATCGTCCAGGTTTGCCAGTTGA-3’, ARM-R, 5’-TGTTCTAGAAAGCTGGCGGCCGCTCAATGATGATGATGATGATGAGCTGGTGGCATCAAAACAGA-3’. The recombinant *arm* construction was amplified from pUC57-ARM plasmid by PCR using PrimeSTAR Max DNA Polymerase (Takara, Japan) at 98°C for 10 s, 55°C for 10 s, and 72°C for 30 s for a total of 30 cycles and purified using TIANgel Purification Kit (TIANGEN, China). *P. pastoris* expression vector pGAPZαA (ThermoFisher, USA) was inserted with the recombinant *arm* PCR product by using ClonExpress^®^ II One Step Cloning Kit (Vazyme, China) after digestion of *EcoR* I and *Not* I. The recombinant plasmids pGAPZαA-ARM were transferred into *E. coli* TOP10 cells (Weidi Biotechnology, China) by heat shock following the manufacture’s protocol. The transferred bacterial cells were cultured on LB plates containing 25 μg/ml Zeocin™ at 37°C overnight. Zeocin™ resistant clones were picked and used as template for colony PCR with primers of the *arm* using PrimeSTAR^®^ Max DNA Polymerase Kit (Takara, Japan). The identified clones with *arm* insertion were inoculated into 10 ml LB medium containing 25 μg/ml Zeocin™ and cultured at 37°C overnight with shaking. The amplified pGAPZαA-ARM was purified using the TIANprep Mini Plasmid Kit (TIANGEN, China). The *P. pastoris* transformation and the ARM expression and purification were performed using pGAPZα A, B, and C Kit (ThermoFisher, USA) following the manufacture’s manual. Briefly, *P. pastoris* GS115 cells were digested with *Avr* II, transformed with pGAPZαA-ARM by electroporation at a charge voltage of 1.5 kV, a capacitance of 25 mF, and a resistance of 200 Ω, and cultured on YPD plates containing 25 μg/ml Zeocin™ at 30°C for 3 days until visible colony formation. The Zeocin™-resistant clones were picked and used as a template for colony PCR with the primers of *arm* using T5 Direct PCR Kit (Plant) (Tsingke Biotechnology, China). The PCR product was analyzed using electrophoresis on 1% agarose gel and Sanger sequencing at Tsingke Biotechnology (Beijing, China). The identified clones were inoculated into 200 ml YPD medium containing 25 μg/ml Zeocin™ and cultured for 60 hours with shaking. The culture medium was collected and applied to Ni-nitrilotriacetic acid metal ion affinity columns (Bio-Rad, USA). After washing with binding buffer containing 0.5 M NaCl, 20 mM Tris-base, pH 7.9 supplemented with 25mM imidazole, the bound ARM was eluted with the binding buffer supplemented with 150 mM imidazole. The ARM protein was confirmed by 12% SDS-PAGE and Western blot with HRP-conjugated anti-His antibody (Proteintech, China) after deglycosylation. The purified ARM protein was aliquoted and stored at -80°C.

### 2.4 Glycosylation analysis and deglycosylation

The model of ARM structure was generated using Robetta server (https://robetta.bakerlab.org/submit.php), and displayed using PyMol program (v2.5, Schrödinger Inc, USA). The glycosylation sites of ARM were analyzed using NetNGlyc (https://services.healthtech.dtu.dk/service.php?NetNGlyc-1.0). De-glycosylation of ARM protein was performed using Glycapeptidase F (Takara Bio, Japan) according to the following protocol. Briefly, 25 μg ARM protein was mixed with 1% SDS in 1 M Tris-HCl buffer (pH 8.6) and heat-denatured at 100°C for 3 min. After cooling, 5 μl of 5% NP-40 was added to neutralize SDS interference. The reaction system was supplemented with 2 μl Glycopeptidase F and adjusted to 25 μl final volume with ddH_2_O, followed by incubation at 37°C for 20 hours. The deglycosylation effect was examined using 12% SDS-PAGE and silver staining (Solarbio, China).

### 2.5 Mice immunization, bacteria challenge, and bacteria load assessment

ARM was diluted in a volume of 100 μl PBS with 30 μg CpG ODN 1826 (InvivoGen, USA) and used for vaccination in doses from 0.01 to 10 μg. Female C57BL/6 mice, aged 4 to 6 weeks, were anesthetized with 1.5% isoflurane, immunized with 100 μl intramuscularly at the posterior thigh three times, 2 weeks apart. Negative control mice received two equivalent doses of CpG ODN. When ARM was used as a BCG booster vaccine, female C57BL/6 mice, aged 4 to 6 weeks, were anesthetized with 1.5% isoflurane, immunized with 1 × 10^6^ CFU BCG in a volume of 100 μl PBS subcutaneously on the back, and/or immunized with 1 or 10 μg ARM plus 30 μg CpG ODN 1826 (InvivoGen, USA) in a volume of 100 μl PBS intramuscularly at the posterior thigh. The CpG ODN was used as an immunization control. Mice were challenged with 5 × 10^6^ CFU *Mtb* H37Ra in a volume of 100 μl PBS via tail vein 4 weeks after boost immunization. Lung and spleen tissue samples from H37Ra challenged mice were collected and homogenized in 1 ml PBS using gentleMACS (Miltenyi, Germany). The suspension of homogenized tissue in three-fold serial dilutions were plated on 7H11 agar plates in a volume of 100 μl each. Plates were cultured at 37°C for 4 weeks and counted for colony forming units (CFU). To assess *Mtb* 16s rRNA copies, the suspension was centrifuged, and the precipitate was resuspended in 1 ml of TRIzol (ThermoFisher, USA). After mixing with 200 μl of chloroform, the aqueous phase was collected after centrifugation and the RNA was precipitated with isopropanol. The RNA pellets were washed with 75% ethanol and resuspended in RNase-free water. The quantity and quality of the RNA product were determined by measuring the OD values at 230, 260 and 280 nm using the RNA program in NanoDrop (ThermoFisher, USA). cDNA was synthesized and genomic DNA was removed using EasyScript^®^ One-Step gDNA Removal and cDNA Synthesis SuperMix (TransGen, China) by mixing 2 μg RNA with the reaction components and incubated at 42°C for 15 minutes, followed by inactivation at 85°C for 5 seconds. Quantitative PCR assessment of *Mtb* 16s RNA copies was performed using the iTaq Universal SYBR Green Kit (Bio-Rad, USA) with primers: 16S-F: 5’-GTGGAGAAGAAGCACCGGC-3’, 16S-R: 5’-ACGCTCGCACCCTACGTATT-3’. The synthesized *Mtb* 16S rRNA (Tsingke Biotechnology, China) with a sequence from NCBI (Gene ID: OR911260.1) and in eight-fold serial dilutions was used as standard for the quantification. Briefly, the cDNA product was mixed with the reaction components following the manufacture’s instruction, and the mixture was loaded onto a CFX Connect Real-Time PCR Detection System (Bio-Rad, USA), and amplified using a recommended thermal cycle program.

### 2.6 ELISA of serum antibody and cytokines

Microwell plates were coated with 100 μl of 2 μg/ml antigen protein in CBS buffer (0.015 mol/L Na_2_CO_3_ and 0.035 mol/L NaHCO_3_, pH=9.6) overnight at 4 °C, blocked with PBS 0.1% Tween-20 and 3% BSA in PBS at 37°C for 2 hours, washed with 0.1% Tween-20 in PBS. Peripheral blood was collected via tail vein of mice. The serum samples 100 μl with an initial dilution of 1:50 followed by 5-fold serial dilution were added into the plate and incubated at 37°C for 1 hour. The plate was washed and incubated with 1:5000 diluted HRP-conjugated anti-IgG, anti-IgG1, or anti-IgG2c antibodies (Proteintech, China) in 0.1% Tween-20 and 0.75% BSA in PBS for 1 hour, and developed with TMB substrate (Solarbio, China) for 5 minutes. The reaction was stopped with 0.5 M H_2_SO_4_, and the optical density was read at 450 nm using a SpectraMax i3x microplate reader (Molecular Devices, USA). The endpoint titer was determined as the highest dilution at which the corresponding OD value was ≥ 2 times that of the negative control. Serum IFN-γ, TNF-α, IL-2, and IL-10 expressions were quantified using mouse cytokine ELISA kits (Yuanju, China) following the manufacture’s instruction. Briefly, 10 μl serum mixed with 40 μl sample diluent were added into wells of 96-well plate coated with anti-IFN-γ, anti-TNF-α, anti-IL-2, or anti-IL-10 monoclonal antibodies respectively, and incubated at 37°C for 1 hour. After washing, 100 μl of HRP conjugated anti-IFN-γ, anti-TNF-α, anti-IL-2, or anti-IL-10 monoclonal antibodies were added to the wells and incubated at 37°C for another hour. The plate was washed and developed with 50 μl of Chromogen Solution A and B at 37°C for 15 min in the dark. After treatment with 50 μl Stop Solution, the optical density was measured at 450 nm using a SpectraMax i3x microplate reader (Molecular Devices, USA).

### 2.7 Flow cytometry assay for cell phenotype and intracellular cytokine expression

Splenic tissue samples were collected from mice, and the single cell suspension was prepared by homogenization using the gentleMACS (Miltenyi, Germany). Mononuclear cells were prepared by using Ficoll (Cytiva, USA) gradient centrifugation, stained with Fixable Viability Stain 700 (BD Biosciences, USA) and fluorochrome-conjugated antibodies CD3-APC-Cy7, CD19-BV421, CD4-BV785, CD8-BV650, CD44-PerCP-Cy5.5, CD62L-BV605, CD69-PE, and CD103-BV510 (BioLegend, USA) at room temperature for 15 min in the dark. After washing and fixation with 2% paraformaldehyde, the cell samples were acquired on FACSymphony™ A5 (BD Biosciences, USA) and the data were analyzed using Flow Jo (v10.6.2, BD FlowJo, USA).

The mononuclear cells at 1 × 10^6^/well were added into a 96-well plate and cultured with 20 μg/ml ARM or 25 μg/ml H37Rv lysate (Gene-Optimal science and technology, China) in the presence of 1 μg/ml anti-CD28/CD49d (eBioscience, USA) at 37°C in 5% CO_2_ for 3 hours. The culture was added with 1 μg/ml GolgiStop (eBioscience, USA) and incubated for another 11 hours. The cells were harvested, washed, and stained with Fixable Viability Stain 700 (BD Biosciences, USA) and fluorochrome-conjugated antibodies CD3-APC-Cy7, CD4-BV605, CD8-BV650, CD44-FITC at room temperature for 15 min in the dark. The stained cells were fixed and permeabilized with Cytofix/Cytoperm™ Fixation/Permeabilization Kit (BD Biosciences, USA) at 4°C for 20 min in the dark. After washing, the cells were stained with anti-INF-γ-BV510, anti-TNF-α-PE-Cy7, and anti-IL-2-BV711 (BioLegend, USA) at room temperature for 15 min in the dark. Cell samples were acquired on FACSymphony™ A5 (BD Biosciences, USA) after washing, and the data were analyzed using Flow Jo (v10.6.2, BD FlowJo, USA).

### 2.8 Histological examination

The left lobe of lung was removed from mice, fixed with buffered 4% polyformaldehyde, embedded in paraffin, sectioned and stained with hematoxylin and eosin (H&E). The slides were scanned using a PANNORAMIC 250 Flash III (3DHISTECH, Hungary), and the photos were taken with the SlideViewer program (2.6.0, 3DHISTECH, Hungary). The level of inflammation present in each image was objectively assessed by the ImageJ program (National Institutes of Health, USA). Briefly, the images were converted into gray scale, and the inflammatory and non-inflammatory areas were marked as black and white, respectively. The inflammatory and non-inflammatory sections on each slide and the percentage of inflammatory sections of the lungs were determined by using the ImageJ program.

### 2.9 Statistical analyses

One-way ANOVA followed by Fisher’s LSD test was used for statistical analysis. *P* values ≤ 0.05 were considered significant.

## 3 Results

### 3.1 The construction and expression of the ARM

**Figure 1:**
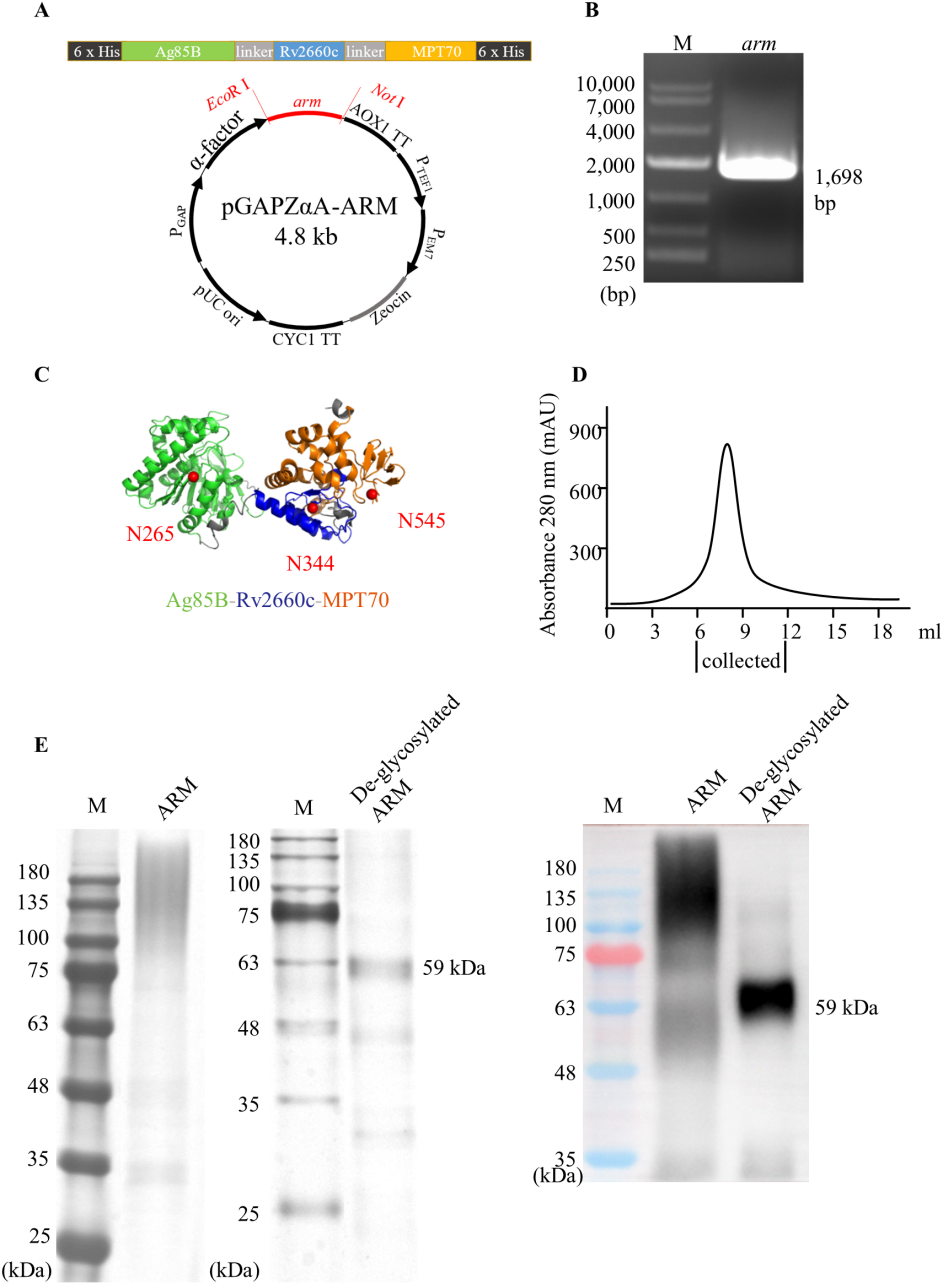
Construction and expression of the ARM. **(A)** Schematic of the *arm* composition, structure, and a recombinant plasmid pGAPZαA-ARM. The *arm* recombinant was inserted into the *P. pastoris* GS115 genome between the *EcoR* I and *Not* I restriction sites. **(B)** The construction and the insertion were verified by agarose gel analysis following PCR amplification of the *arm* insert. M: molecular mass markers. **(C)** The structural illustration of the ARM was generated using the Robetta server online service and displayed using the PyMol program. The Ag85B, Rv2660c, and the MPT70 are represented by green, blue, and orange color codes respectively. Glycosylation of the ARM was predicted using the NetNGlyc-1.0 server online service. Three *N*-glycosylation sites, N265, N344, and N545 were predicted on the Ag85B, Rv2660c, and MPT70 regions, respectively, indicated by solid red circles. **(D)** Elution profile of ARM protein from a Ni-nitrilotriacetic acid metal ion affinity columns. **(E)** The purified ARM was treated with glycopeptidase F and loaded onto SDA-PAGE for electrophoresis analysis. The band of ARM with a molecular mass of 59 kDa was identified by silver staining (left panel) or by Western blot (right panel) probed with HRP-conjugated anti-His monoclonal antibody.

The nucleic acids encoding the *ag85B*, *rv2660c*, and *mpt70* segments were linked in tandem to generate a recombinant *arm*. Two 6 × *his* tags were added to the front and back ends of the recombinant *arm*. The recombinant *arm* construct was ligated into the pGAPZαA plasmid between the *EcoR* I and *Not* I restriction enzyme sites to generate a recombinant plasmid pGAPZαA-ARM (**Figure 1A**). After transformation with the plasmid pGAPZαA-ARM, *P. pastoris* GS115 cells were plated and cultured on YPD plates containing the antibiotic Zeocin. The Zeocin-resistant colony was analyzed by PCR and Sanger sequencing to identify the 1,698 bp *arm* construction (**Figure 1B**). Glycosylation of the ARM protein were analyzed using NetNGlyc-1.0. Three N-glycosylation sites, N265, N344, and N545, were identified on Ag85B, Rv2660c, and MPT70 segments respectively (**Figure 1C**). The identified clones were inoculated into YPD medium and cultured for 60 hours with shaking. The culture medium was applied to Ni-nitrilotriacetic acid metal ion affinity columns for isolation and purification of the ARM, and a major elution peak was recovered (**Figure 1D**). The glycosylated ARM protein showed a smeared band above the 59 kDa predicted mass on SDS-PAGE, most likely due to multiple glycoforms with different sugar chain lengths and compositions, variations in the charge-to-mass ratio, altered protein conformation due to hydrophilicity, and aggregation with carbohydrates. This assumption was confirmed by anti-His antibody binding on the Western blot (**Figure 1E**). The gray areas on the SDS-PAGE were quantitatively analyzed using ImageJ, which showed a purity of 94% for the purified ARM protein. Subsequent de-glycosylation with glycopeptidase F yielded a predominant 59 kDa band on both SDS-PAGE and Western blot, which confirmed the glycan modification by *P. pastoris*. Notably, this de-glycosylated ARM band marginally exceeded the theoretical mass. This minor discrepancy could be attributed to either residual glycosylation or imperfect prediction of the molecular mass.

### 3.2 ARM inoculation induced an antigen-specific immune response and reduced the bacterial load in mice challenged with *Mtb* H37Ra

**Figure 2:**
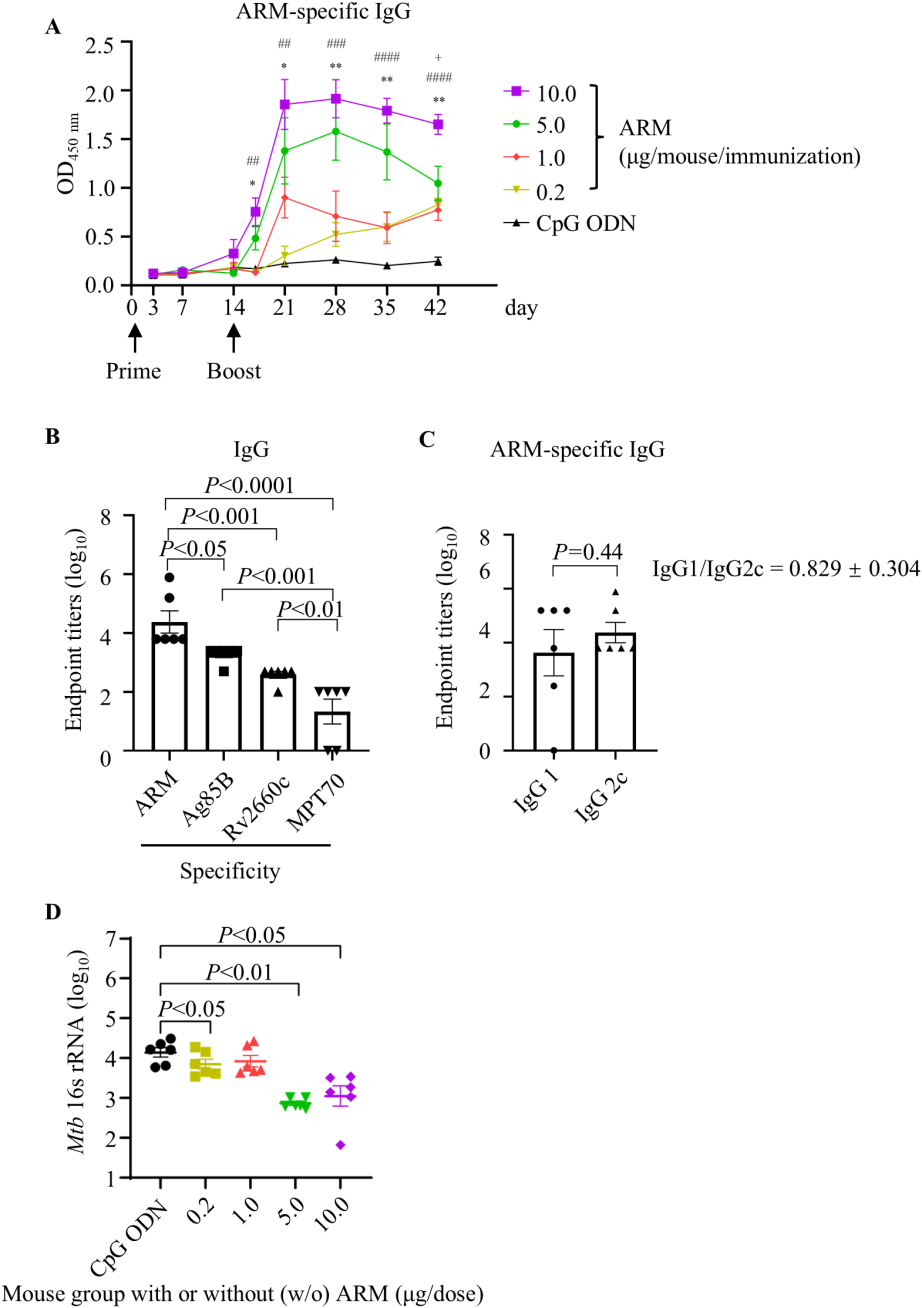
ARM-induced antibody response and the reduced bacterial load in ARM-immunized mice after *Mtb* challenge. **(A)** C57BL/6 mice were primed and boosted intramuscularly with different doses of ARM, two weeks apart. Peripheral blood was collected via the tail vein at the indicated time intervals, and the ARM-specific IgG in serum was assessed by ELISA. Data are from one experiment with six mice per group. ^*^ or ^+^ *P* < 0.05, ^**^ or ^##^ *P* < 0.01, ^###^ *P* < 0.001, ^####^ *P* < 0.0001 by two-way ANOVA followed by Fisher’s LSD test. ^*^ represents statistical differences between CPG ODN group and 5.0 μg/dose group, ^#^ represents statistical differences between CPG ODN group and 10.0 μg/dose group, ^+^ represents statistical differences between 5.0 μg/dose group and 10.0 μg/dose group. **(B)** Serum samples from the 10.0 μg/dose group were evaluated for IgG titers against Ag85B, Rv2660c, and MPT70 respectively at week 4 post boost. **(C)** Serum samples from the 10.0 μg/dose group were evaluated for ARM-specific IgG subclass at 4 weeks post boost. **(D)** Mice were challenged intravenously with *Mtb* H37Ra strain 4 weeks post boost. Lung tissues were collected at week 5 post challenge for RNA isolation. The copies of *Mtb* 16s rRNA in the lungs were examined by real-time RT-PCR. Data are from one experiment with six mice per group. One-way ANOVA followed by Fisher’s LSD test was used for statistical analysis.

To evaluate the immunogenicity of ARM, C57BL/6 mice were primed intramuscularly with different doses of the ARM with CpG ODN as adjuvant and boosted with the same regimen respectively 2 weeks after priming. Mice were bled via tail vein at the indicated intervals for serum collection. The ARM-specific IgG was assessed by ELISA. Levels of ARM-specific IgG elevated approximately 7 days post the prime and increased significantly after the boost. In 5.0 μg/dose and 10.0 μg/dose groups, antibody levels peaked at day 7 post boost and maintained for approximately another 7 days, then began to decline. In 0.2 μg/dose group, the ARM-specific IgG showed a mild elevation after boost but continued to increase until day 42. No serum IgG was detected in mice inoculated with CpG ODN as a control. The ARM-specific IgG levels correlated with the inoculation dose. The ARM 10.0 μg/dose group showed the highest level of ARM-specific IgG in serum (**Figure 2A**). Quantitatively assessing the antibody with respective specificities, the Ag85B-specific IgG had the highest and the MPT70-specific IgG showed the lowest level, while the Rv2660c-specific IgG level was in between (**Figure 2B**). Studying sub-class of the ARM-specific IgG using anti-Ig antibodies revealed that the IgG2c level was slightly higher than the IgG1 level (**Figure 2C**). The IgG1/IgG2 ratio was 0.829 ± 0.304.

Mice were challenged with *Mtb* H37Ra via tail vein after 4 weeks of boost, and the lung tissues were collected after 5 weeks of challenge for the evaluation of bacterial burden by absolute quantitative PCR assessing the copies of *Mtb* 16s rRNA, a rapid and specific assessment. Mice immunized with ARM had significantly fewer copies of *Mtb* 16s rRNAs than those immunized with CpG ODN. Among those immunized with ARM, the 5.0 μg/dose and 10.0 μg/dose groups had significantly fewer copies of *Mtb* 16s rRNA than the 0.2 μg/dose and 1.0 μg/dose groups. There was no difference in *Mtb* 16s rRNA copies between the 5.0 μg/dose and 10.0 μg/dose groups and between the 0.2 μg/dose and 1.0 μg/dose groups (**Figure 2D**). Since immunization with 10.0 μg/dose ARM induced the highest level of ARM-specific IgG and significantly reduced the *Mtb* H37Ra burden, we used this dose for the following experiments.

### 3.3 ARM boost promoted multi-functional *Mtb*-specific CD4^+^ T cells in BCG-primed mice

**Figure 3:**
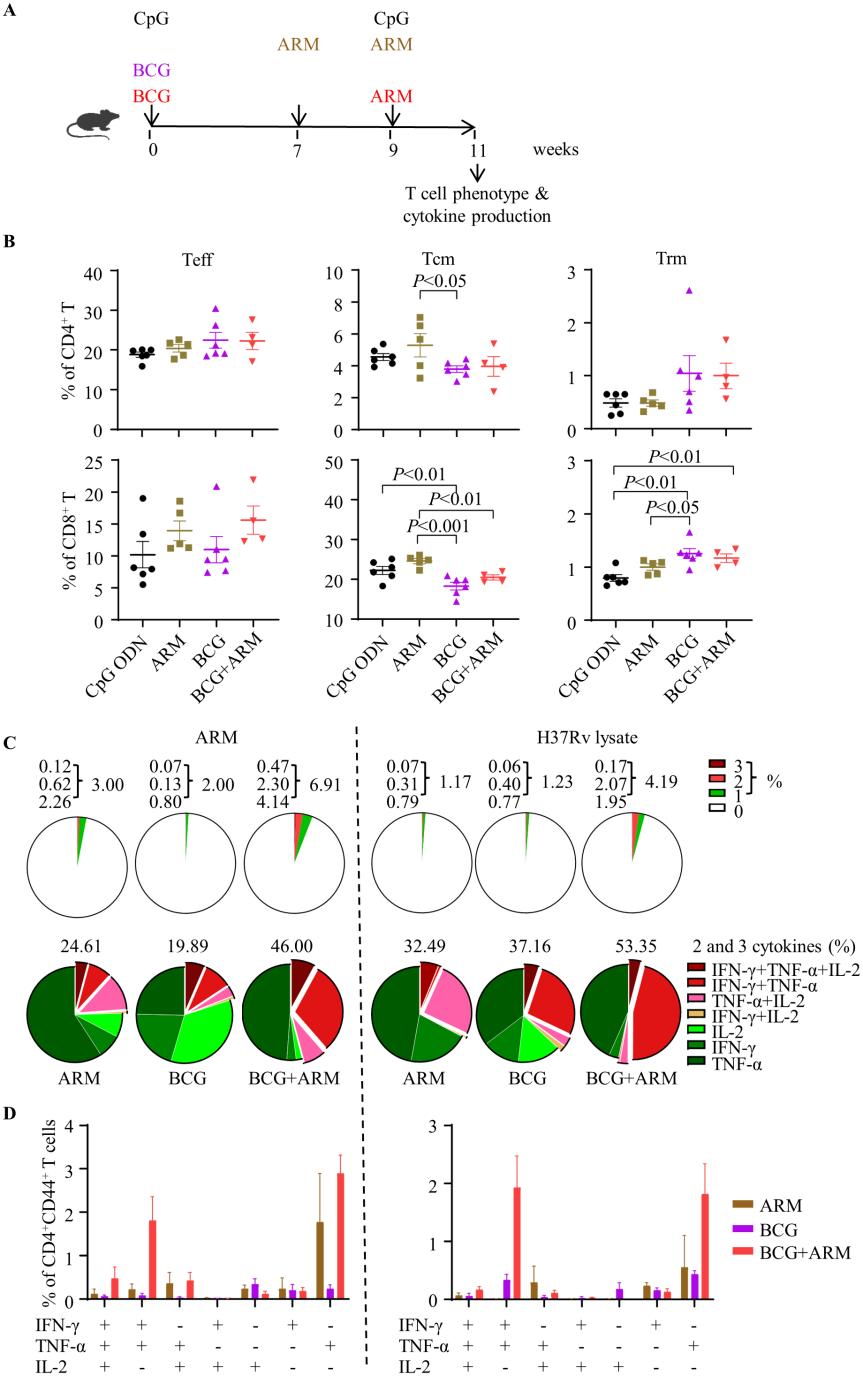
T cell differentiation and multi-cytokine production promoted by ARM boost in BCG-immunized mice. **(A)** Schematic of the immunization schedule. C57BL/6 mice were primed with BCG subcutaneously or ARM and CpG ODN intramuscularly and boosted with ARM (10 μg) and CpG ODN intramuscularly. Adjuvant CpG ODN was used as control. **(B)** Spleens were isolated for preparation of mononuclear cell suspension at week 11 post the prime. The differentiation phenotype (effector, central memory, and tissue resident T cells, represented by Teff, Tcm, and Trm, respectively) of the splenic T cells was analyzed with flow cytometry. Data are representative of two independent experiments, each with four to six mice per group, and analyzed with one-way ANOVA followed by Fisher’s LSD test. **(C)** Spleen cells were stimulated with the ARM or H37Rv lysate in the presence of brefeldin A and analyzed for intracellular cytokine expression of CD4^+^CD44^+^ T cells with flow cytometry. The proportion of cytokine-expressing cells in the total CD4^+^CD44^+^ T cell population (upper panel) and the proportion of cells expressing one, two, or three cytokines in the cytokine-producing CD4^+^CD44^+^ T cell population (lower panel) were shown in pie charts. Data are representative of two independent experiments, each with three to four mice per group. **(D)** The proportions of cells producing a combination of 3 cytokines, 2 cytokines, and 1 cytokine only were shown. Data are representative of two independent experiments, each with three to four mice per group.

The schedule for the immunization and the assessment of the T cell differentiation and cytokine production is shown in **Figure 3A**. Spleen tissues were collected at week 11 for mononuclear cell preparation, and the phenotype of bulk T cells and cytokine production induced by *Mtb* antigens were assessed by flow cytometry. The gating strategy was shown as **Supplementary Figure S1A**. BCG prime with or without ARM boost reduced the frequency of central memory T cells (Tcm) expressing CD44^+^CD62L^+^ but increased the frequency of tissue resident T cells (Trm) expressing CD69^+^CD103^+^ in both CD4^+^ and CD8^+^ T cell populations compared to CpG ODN control and ARM only (**Figure 3B**).

Spleen mononuclear cells were stimulated with ARM or *Mtb* H37Rv lysate *in vitro*, and stained for phenotyping and expressing IFN-γ, TNF-α, and IL-2 intracellularly (**Supplementary Figure S1B**). The frequencies of cells expressing single and multiple cytokines in the CD4^+^CD44^+^ T cell population were shown in a pie chart (**Figure 3C**). BCG prime with ARM boosting increased the frequency of cytokine-producing cells in response to both ARM and H37Rv lysate stimulation *in vitro* in compared to BCG or ARM alone (**Figure 3C**, top panel). Among these cytokine-producing cells, ARM boosting significantly increased the cells producing 2 or more cytokines, whereas BCG or ARM only increased cells producing one cytokine (**Figure 3C**, bottom panel). The ARM boosting mainly increased cells producing a combination of 3 cytokines, a combination of IFN-γ and TNF-α, a combination of TNF-α and IL-2, and cells expressing TNF-α only (**Figure 3D**).

### 3.4 ARM boost reduced bacterial CFU upon *Mtb* challenge in BCG-primed mice

**Figure 4:**
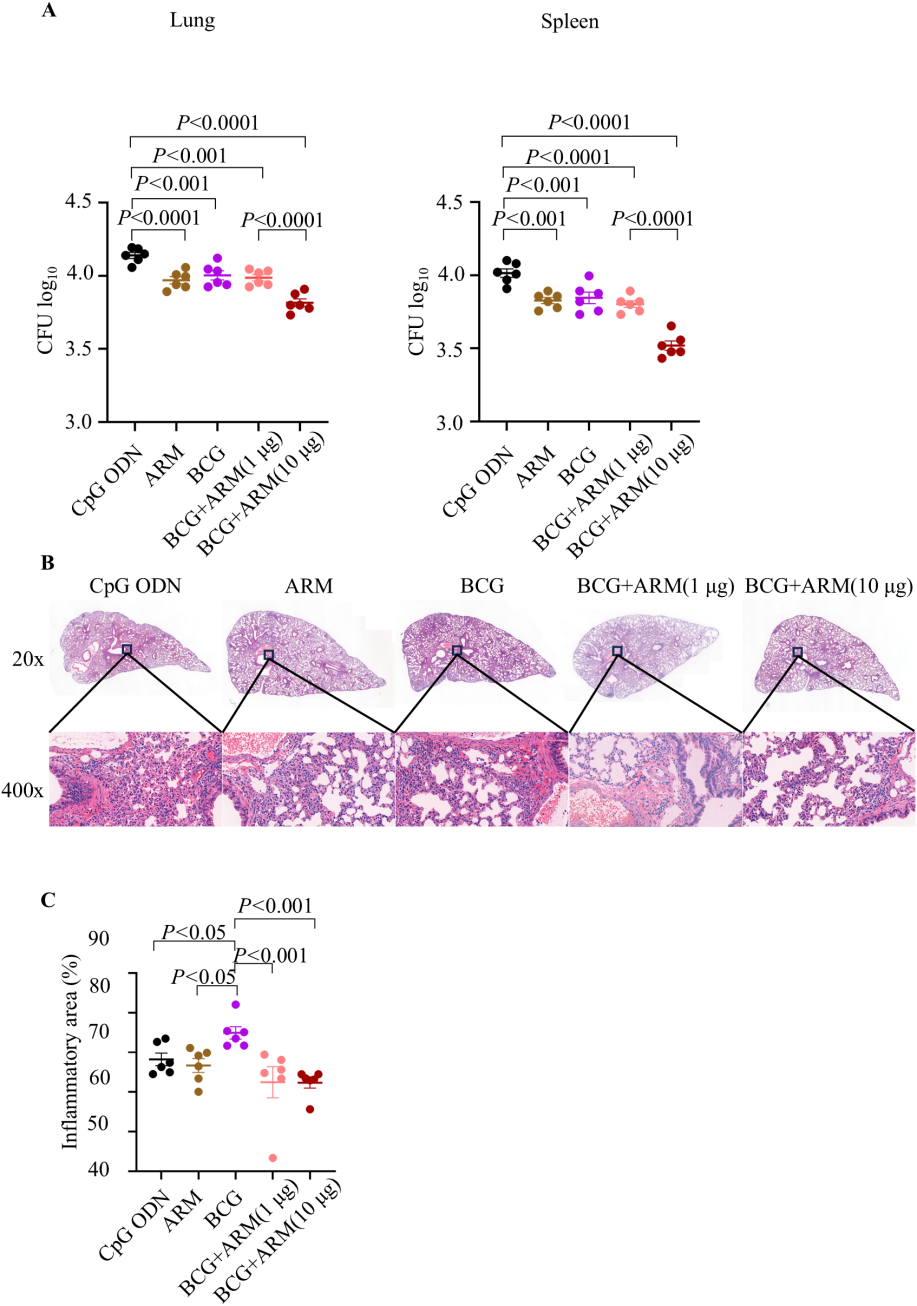
Bacterial load and lung inflammation after *Mtb* challenge in BCG-primed and ARM-boosted mice. C57BL/6 mice were primed with ARM or BCG, boosted with or without ARM (in 1 and 10 μg), and challenged with H37Ra intravenously at week 4 post boost. **(A)** Lung and spleen tissues were collected at week 5 post challenge and homogenized in PBS. The homogenized suspensions were plated on 7H11 agar plates in a series of three-fold dilutions and counted for CFUs 4 weeks later. Data are representative of two independent experiments, each with six mice per group, and analyzed with one-way ANOVA followed by Fisher’s LSD test. **(B)** Lung tissues were fixed in 4% paraformaldehyde, embedded in paraffin, sectioned and stained with hematoxylin and eosin. The tissue structure and cell infiltration were studied under a PANNORAMIC 250 Flash III. The sections shown are representative of six animals per group. **(C)** The inflammatory area of lung was estimated from microscopically scanned images and analyzed using the ImageJ. Data are representative of two independent experiments, each with six mice per group, and analyzed with one-way ANOVA followed by Fisher’s LSD test.

Mice were immunized as proposed in **Figure 3A** and challenged with *Mtb* H37Ra strain vial tail vein at week 4 post boost. Lung and Spleen tissues were collected after 5 weeks of *Mtb* challenge, and the bacterial load was evaluated by counting the CFU in tissue culture. The ARM prime and boost, BCG only, and BCG prime with ARM boost (in 1 and 10 μg) all reduced *Mtb* CFU in the lung and spleen compared to the CpG ODN control. Among these reductions, the BCG prime with 10 μg ARM boost group had the lowest CFU counts, while the ARM prime and boost, BCG only, and the BCG prime with 1 μg ARM boost groups had relatively small scale but significant reductions (**Figure 4A**).

Histology of the lung sections, collected at week 5 post *Mtb* challenge and stained with hematoxylin and eosin, shows that BCG prime with ARM boost reduced the pathological inflammation in mice (**Figure 4B**). The lung sections from CpG ODN and ARM immunized mice showed a magnitude of inflammatory infiltration with mostly polymorphonuclear leukocytes and mononuclear cells, peribronchiolar and pericapillary aggregation of infiltrates, interstitial thickening with infiltrates present, destructed structure of alveoli, and epithelial necrosis with pyknotic nucleus. Although sections from BCG immunization group showed reduced inflammatory infiltration and infiltrates aggregation, the interstitial spaces were presented with polymorphonuclear leukocytes and mononuclear cells, and the alveolar structures were damaged. The sections from BCG prime with ARM boost mice show significantly less inflammatory infiltration, fewer or no infiltrate aggregates, few infiltrates in interstitium with relatively intact of alveolar structure compared to sections from the CpG ODN, ARM, and BCG groups. The inflammatory responses can be compared quantitatively by examining the area of the inflammatory lesion. We found that the lungs of the BCG prime group had more inflammatory areas after the *Mtb* H37Ra challenge. But both BCG + ARM (1 μg) and BCG + ARM (10 μg) groups showed significantly less inflammation compared to the BCG-only group. However, these reductions were not significant when compared to the adjuvant control group and the ARM prime-and-boost group (**Figure 4C**).

### 3.5 ARM boost induced persistent IFN-γ but diminished IL-10 expressions upon *Mtb* challenge in BCG-primed mice

**Figure 5:**
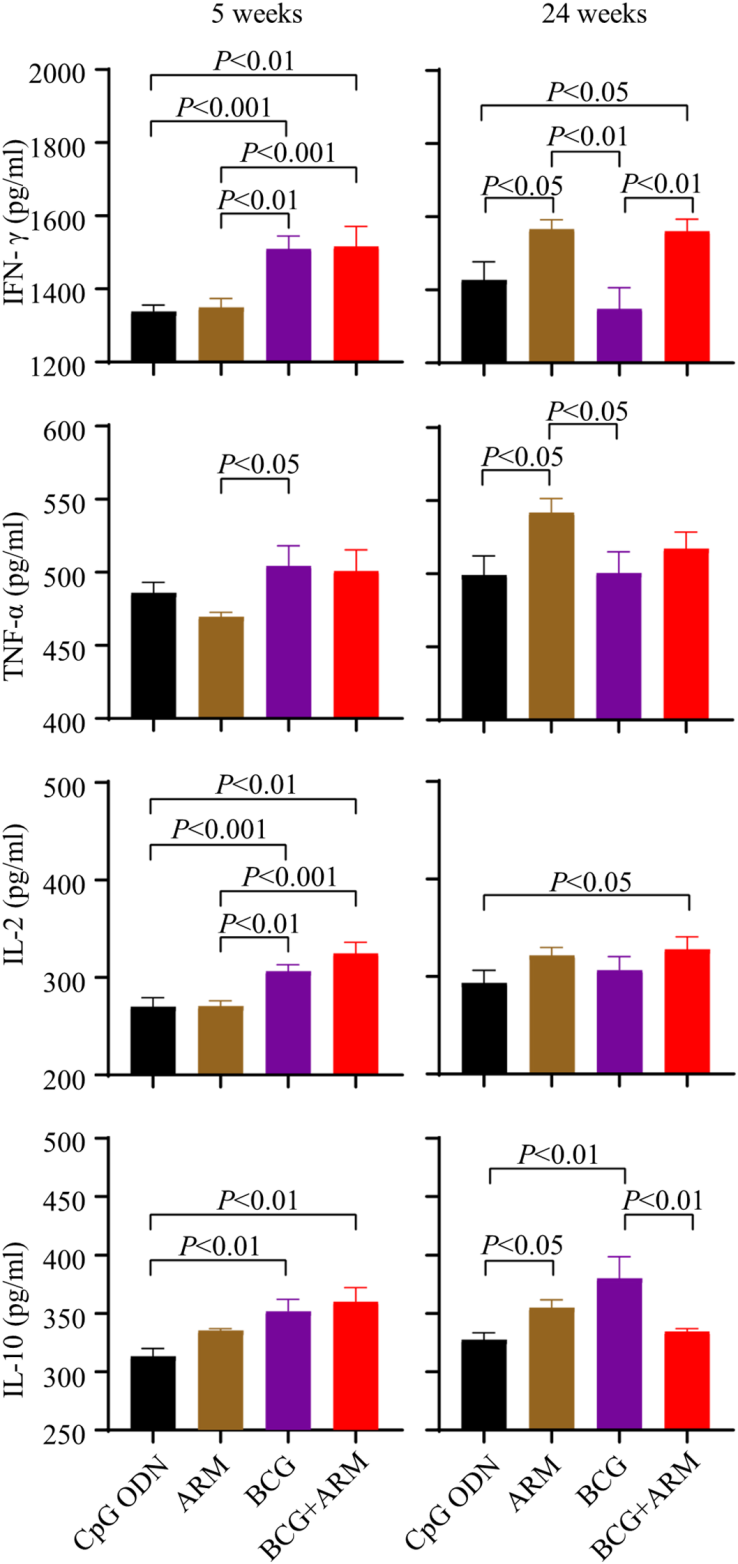
Magnitude and persistence of cytokine expression upon *Mtb* challenge in BCG-primed and ARM-boosted mice. C57BL/6 mice were primed with ARM or BCG, boosted with or without ARM (10 μg), and challenged with H37Ra intravenously at week 4 post boost. Peripheral blood was collected at week 5 and 24 post the challenge. The concentrations of IFN-γ, TNF-α, IL-2, and IL-10 in serum were assessed using ELISA. Data are from one experiment, each with four to six mice per group, and analyzed with one-way ANOVA followed by Fisher’s LSD test.

Mice were primed and boosted as proposed in **Figure 3A** and challenged with *Mtb* H37Ra strain via tail vein at week 4 post boost. Serum samples were collected at week 5 and 24 post challenge. The concentrations of IFN-γ, TNF-α, IL-2, and IL-10 in the serum of the mice were assessed by ELISA. BCG prime with or without ARM boost both increased the IFN-γ, TNF-α, IL-2, and IL-10 levels compared to ARM alone and CpG ODN control at week 5 post challenge. However, BCG prime with ARM boost maintained the elevated IFN-γ for at least 24 weeks after challenge, while the BCG only group had a diminished IFN-γ relative to the level at week 5 post challenge. The BCG prime with ARM boost also maintained an elevated IL-2 relative to the CpG ODN control for at least 24 weeks after challenge. The BCG prime with ARM boost group showed a diminished IL-10 at week 24 post challenge, while the BCG only group maintained or slightly increased IL-10 levels at week 24 post challenge (**Figure 5**).

## 4 Discussion

In this study, we designed a recombinant protein ARM expression construct containing antigenic components Ag85B, Rv2660c, and MPT70 that are shared by *Mtb* and BCG, and expressed this glycosylated fusion protein using a *P. pastoris* GS115 expression system. The ARM immunization induced antibody production against all three fusion components in mice and facilitated H37Ra reduction upon the pathogen challenge. ARM boosting prompted BCG-induced CD4^+^ T cell response with increased Th1-type cytokine production and proportion of multifunctional CD4^+^ T cells and facilitated the H37Ra reduction without significant lung inflammation. The ARM augmentation also prolonged the elevated level of IFN-γ presence in serum in response to H37Ra challenge in BCG-primed mice. The result demonstrated that the ARM improved the quality of BCG-induced immune response, increased its potency in pathogen reduction and prolonged the infection-induced effector cytokine production. The data suggest that the ARM has the potential to improve the efficacy of BCG.

The rational design of the ARM was based on reports that immunization with Ag85B or MPT70 enhanced Th1 responses against *Mtb* and facilitated the *Mtb* reduction in mice (11, 15). Vaccination with Rv2660c in combination with Ag85B-ESAT-6 induced antigen-specific Th1-type immunity, reduced the *Mtb* burden, and suppressed LTBI reactivation (7). In constructing the ARM expression, we used flexible linkers to bridge the genes encoding Ag85B, Rv2660c, and MPT70. The flexible linkers were used as insulators between these coding sequences to mitigate potential misfolding of the antigen proteins and to ensure the immunogenicity of individual component (30). Glycosylation is also critical to protein immunogenicity. Ag85B is glycosylated proteins expressed by *Mtb* (25) and the consensus *N*-glycosylation sites, Asn-X-Ser/Thr, are at residues N265, N344, and N545 on Ag85B, Rv2660c, and MPT70, respectively. We used *P. pastoris* GS115 system to express the constructed ARM because this system has a glycosylation pattern similar to *Mtb*. For instance, Rv1002c from *Mtb* and Pmt1p from *P. pastoris*, as *O*-mannosyltransferases, are high homologous. Consequently, the Ala-Pro-rich region expressed by *P. pastoris* displays a mannosylation pattern comparable to that expressed by *Mtb* (28). We used the plasmid pGAPZαA as a vector to introduce the arm into the yeast cells. The α-factor signal sequence renders the constitutive expression of the 59 kDa ARM protein in a secretive form (31). This glycosylated ARM induced antibody production in mice in a dose dependent manner. Among the antibodies produced, anti-Ag85B was the most abundant, followed by anti-Rv2660c, and the anti-MPT70 was the least abundant. The disproportionate expression might be due to the MHC class II binding affinity of the epitopes and the frequency of precursor of epitope specific T cells. The Ag85B-Rv2875-Rv3478-Rv3619 fusion protein induced a dominant Ag85B-specific CD4^+^ T cell response in mice, whereas the MPT70 (Rv2875)-specific CD4^+^ T cell response was subdominant (16). Our result demonstrates that we have successfully constructed an ARM expression system. The system expresses ARM in glycosylated form in secretive way. The ARM is immunogenic and retains their antigenicity of individual components, inducing antibody responses to the cognate antigens.

ARM-inoculated mice had significantly lower H37Ra 16s rRNA loads than those with adjuvant alone, and the reduction was ARM dose dependent. Although T cell response induced by the ARM also played a role, we believe that the antibodies were the major effector molecule in this reduction. Our data showed that ARM immunization did not significantly promote T cell activation nor differentiation, did not increase the proportion of cytokine production of T cells. The protective effect of *Mtb*-specific antibody has not been fully recognized, and the mechanism of protection has not been depicted in detail. However, *Mtb*-specific antibodies have been shown to contribute to anti-TB protection (20). IgG against mycobacterial capsular polysaccharide arabinomannan promoted FcγR-mediated phagocytosis and intracellular growth inhibition of *Mtb* (32). PPD-specific IgG enhanced natural killer cell-mediated cytotoxicity to *Mtb* (33). The mounting evidence and our data support that the ARM immunization induces specific B cell response and the induced antibody production play a role in the protective immunity against *Mtb* infection.

The ARM is also potent to promote the BCG-induced immune response and reduce CFU in mice following *Mtb* challenge. Our data showed that mice primed with BCG and boosted with 10 µg ARM had increased cytokine producing T cells relative to those without ARM, particularly CD4^+^ T cells producing IFN-γ, TNF-α and IL-2 Th1 cytokines and the proportion of multi-cytokine producing cells. In our observation, most T cells responding to the BCG prime with or without 10 µg ARM boost are CD4^+^ T cells. This is consistent with the observation that CD4^+^ T cells are the most prominent protective cells against TB (34). Th1 cytokines are essential to support T cell activation, proliferation, and differentiation, and to provide effector function to eliminate pathogens, and to build immune memory to establish a posture to mount a sufficient effector response upon antigen encounter to eliminate pathogens early in the infection (35). The T cell activation and differentiation are also essential for CD4^+^ T cells to support B cell antibody production and class switching (36, 37). The ARM immunization increased the proportion of multi-functional CD4^+^ T cells. T cells producing 2 or more cytokines have been reported to be more efficient in *Mtb* eradication (38). The ARM boost did not significantly alter the T cell activation and differentiation. The proportion of effector T cells in the CD4^+^ and CD8^+^ T cell populations and the cytokine-producing CD4^+^ T cells were not significantly elevated. Uncontrolled inflammatory immune responses, such as granulocytic influx, have been reported to be associated with immune-mediated tissue damage (39). Mice with ARM boost showed less pulmonary immunopathology compared to those with BCG alone. The ARM boost enhanced immune response facilitated *Mtb* reduction by promoting the quality other than the quantity of T cell response, and by enhancing both the B and T cell responses other than T cell response alone.

Our data showed that mice with 10 µg ARM boost had a lower level of IL-10 but higher levels of IFN-γ in serum 24 weeks after challenge. IL-10 was reported as an anti-inflammatory cytokine that promoted the persistence of *Mtb* (40), and the increase of IL-10 at the end of chemotherapy were considered as a risk factor for TB recurrence (41). BCG-induced IL-10 inhibited the production of S100A8/A9, thereby inhibiting IFN-γ production by CD4^+^ T cells and hindering the development of effector memory Th1 cells (42). Our observation suggests a durable protective immunity in 10 µg ARM boosted mice.

Our study has limitations. (1) Due to the constrain of laboratory biosafety, we used mouse model and H37Ra intravenous challenge to test our proposal of using recombinant fusion protein to enhance the anti-*Mtb* immunity induced by BCG and to improve BCG efficacy. Although a virulent strain and aerosol challenge are preferred to evaluate the efficacy of immunization against *Mtb* infection, the H37Ra and intravenous challenge are widely used to evaluate the immune response induced by *Mtb* or by vaccination (43). But this approach does not necessarily demonstrate the ARM’s ability of enhancing BCG-induced protection against *Mtb* virulent strain. The conclusion drawn from this study should be further tested and confirmed in non-human primate model with H37Rv strain via airway challenge model; (2) We used all-female mice in this study in anticipation of a strong antibody response and to make our data comparable to other reports using the female mouse model. The data and interpretation may need to be reviewed for applicability to male mice. (3) Although our data suggested that 10 µg AMR boosting improved the quality of T cell response induced by BCG priming, we were unable to identify *Mtb*-specific T cells nor to study their phenotypes due to the lack of *Mtb* epitope-MHC probe. The profiles of Ag85B-, Rv2660c-, and MPT70-specific T cells, including their activation, proliferation, and differentiation, could be enriched by using fluorescence-conjugated epitope-MHC complex in the content of related signature markers. We also acknowledge that the ARM-specific and *Mtb*-specific immune responses in the lungs have not been fully addressed, which will be our following exploration. Further addressing these remaining questions will support the development of a better proposal to improve the BCG efficacy.

## Data Availability

The raw data supporting the conclusions of this article will be made available by the authors, without undue reservation.

## Supplementary material

Supplementary Figure 1 Gating strategy for flow cytometry analysis. Spleens were isolated for preparation of mononuclear cell suspension at week 11 post the prime. Singlets of the mononuclear cell preparation were distinguished by FSC area and FCS height. The lymphocyte population was distinguished by FSC area and SSC area. T cells were gated on live and CD3 positive population. From CD4 and CD8 T cell population, **(A)** the Teff, Tcm, and Trm cells were identified by CD44^+^ & CD62L^−^, CD44^+^ & CD62L^+^, and CD69^+^ & CD103^+^, respectively. **(B)** The cytokine-producing CD4^+^ & CD44^+^ T cell in response to ARM and H37Rv lysate were identified by anti-IFN-γ, TNF-α, and IL-2 respectively.
