# Supplementary figures and images for: Recombinant protein Ag85B-Rv2660c-MPT70 promotes quality of BCG-induced immune response against Mycobacterium tuberculosis H37Ra
Source: Front Immunol. 2025 Mar 13;16:1430808. doi: 10.3389/fimmu.2025.1430808 (PMC11965932; doi:10.3389/fimmu.2025.1430808)

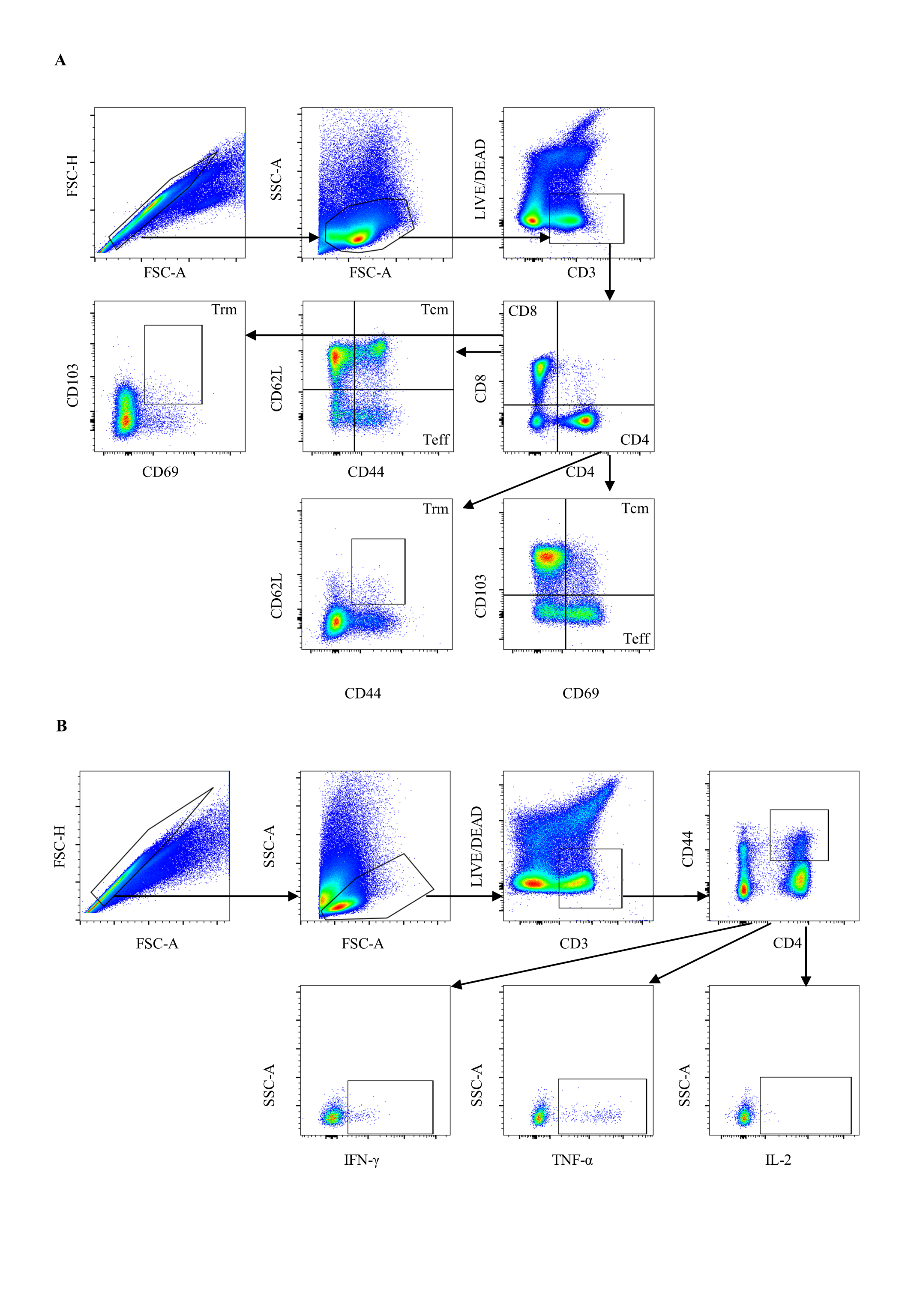

Supplement: Supplementary Figure 1 — Gating strategy for flow cytometry analysis. Spleens were isolated for preparation of mononuclear cell suspension at week 11 post the prime. Singlets of the mononuclear cell preparation were distinguished by FSC area and FCS height. The lymphocyte population was distinguished by FSC area and SSC area. T cells were gated on live and CD3 positive population. From CD4 and CD8 T cell population, (A) the Teff, Tcm, and Trm cells were identified by CD44+ & CD62L−, CD44+ & CD62L+, and CD69+ & CD103+, respectively. (B) The cytokine-producing CD4+ & CD44+ T cell in response to ARM and H37Rv lysate were identified by anti-IFN-γ, TNF-α, and IL-2 respectively. [file Image1.tif]
